# Automated detection of frog calls and choruses by pulse repetition rate

**DOI:** 10.1111/cobi.13718

**Published:** 2021-05-07

**Authors:** Sam Lapp, Tianhao Wu, Corinne Richards‐Zawacki, Jamie Voyles, Keely Michelle Rodriguez, Hila Shamon, Justin Kitzes

**Affiliations:** ^1^ Department of Biological Sciences University of Pittsburgh Pittsburgh Pennsylvania USA; ^2^ Department of Biology University of Nevada, Reno Reno Nevada USA; ^3^ Smithsonian Conservation Biology Institute National Zoological Park Front Royal Virginia USA

**Keywords:** acoustic, amphibian, classification, detection, endangered, machine learning, monitoring, signal processing, acústico, anfibio, aprendizaje mecánico, clasificación, detección, en peligro, monitoreo, procesamiento de señal

## Abstract

Anurans (frogs and toads) are among the most globally threatened taxonomic groups. Successful conservation of anurans will rely on improved data on the status and changes in local populations, particularly for rare and threatened species. Automated sensors, such as acoustic recorders, have the potential to provide such data by massively increasing the spatial and temporal scale of population sampling efforts. Analyzing such data sets will require robust and efficient tools that can automatically identify the presence of a species in audio recordings. Like bats and birds, many anuran species produce distinct vocalizations that can be captured by autonomous acoustic recorders and represent excellent candidates for automated recognition. However, in contrast to birds and bats, effective automated acoustic recognition tools for anurans are not yet widely available. An effective automated call‐recognition method for anurans must be robust to the challenges of real‐world field data and should not require extensive labeled data sets. We devised a vocalization identification tool that classifies anuran vocalizations in audio recordings based on their periodic structure: the repeat interval‐based bioacoustic identification tool (RIBBIT). We applied RIBBIT to field recordings to study the boreal chorus frog (*Pseudacris maculata*) of temperate North American grasslands and the critically endangered variable harlequin frog (*Atelopus varius*) of tropical Central American rainforests. The tool accurately identified boreal chorus frogs, even when they vocalized in heavily overlapping choruses and identified variable harlequin frog vocalizations at a field site where it had been very rarely encountered in visual surveys. Using a few simple parameters, RIBBIT can detect any vocalization with a periodic structure, including those of many anurans, insects, birds, and mammals. We provide open‐source implementations of RIBBIT in Python and R to support its use for other taxa and communities.

## Abstract 2

*Article impact statement*: The repeat interval‐based bioacoustic identification tool automatically identifies frog calls in field recordings.

## Resumen

Los anuros (ranas y sapos) se encuentran dentro de los grupos taxonómicos más amenazados a nivel mundial. La conservación exitosa de los anuros dependerá de información mejorada sobre el estado y los cambios en las poblaciones locales, particularmente para las especies raras y amenazadas. Los sensores automatizados, como las grabadoras acústicas, tienen el potencial para proporcionar dicha información al incrementar masivamente la escala espacial y temporal de los esfuerzos de muestreo poblacional. El análisis de dicha información requerirá herramientas robustas y eficientes que puedan identificar automáticamente la presencia de una especie en las grabaciones de audio. Como las aves y los murciélagos, muchas especies de anuros producen vocalizaciones distintivas que pueden ser capturadas por las grabadoras acústicas autónomas y también son excelentes candidatas para el reconocimiento automatizado. Sin embargo, a diferencia de las aves y los murciélagos, todavía no se cuenta con una disponibilidad extensa de herramientas para el reconocimiento acústico automatizado de los anuros. Un método efectivo para el reconocimiento automatizado del canto de los anuros debe ser firme ante los retos de los datos reales de campo y no debería requerir conjuntos extensos de datos etiquetados. Diseñamos una herramienta de identificación de las vocalizaciones: la herramienta de identificación bioacústica basada en el intervalo de repetición (RIBBIT), el cual clasifica las vocalizaciones de los anuros en las grabaciones de audio con base en su estructura periódica. Aplicamos la RIBBIT a las grabaciones de campo para estudiar a dos especies: la rana coral boreal (*Pseudacris maculata*) de los pastizales templados de América del Norte y la rana arlequín variable (*Atelopus varius*), críticamente en peligro de extinción, de las selvas tropicales de América Central. Mostramos que RIBBIT puede identificar correctamente a las ranas corales boreales, incluso cuando vocalizan en coros con mucha superposición, y puede identificar las vocalizaciones de la rana arlequín variable en un sitio de campo en donde rara vez se le ha visto durante censos visuales. Mediante relativamente unos cuantos parámetros simples, RIBBIT puede detectar cualquier vocalización con una estructura periódica, incluyendo aquellas de muchos anuros, insectos, aves y mamíferos. Proporcionamos implementaciones de fuente abierta de RIBBIT en Python y en R para fomentar su uso para otros taxones y comunidades.

## Introduction

Globally, many anurans (frogs and toads) are in danger of extinction (Scheele et al. 2019). Over 2000 (25%) of the approximately 7900 known amphibian species are classified as vulnerable, endangered, or critically endangered (IUCN 2020), and over 1000 of these threatened species are categorized as data deficient (González‐del‐Pliego et al. 2019). In response, several authors have suggested that biologists need to significantly expand anuran monitoring in order to prevent dramatic biodiversity loss (Young et al. 2001; Storfer 2003; Collins & Halliday 2005). Traditional anuran monitoring approaches include both visual encounter and acoustic surveys by trained human observers (Dodd 2010). In practice, however, logistical and financial limitations lead to trade‐offs between the number of sites sampled and amount of time spent at each site (Dorcas et al. 2009). For example, many acoustic survey protocols use 5‐min listening periods (Dorcas et al. 2009), even though longer listening periods could increase the biodiversity observed (Pierce & Gutzwiller 2007).

Autonomous recording units (Hill et al. 2019) can expand monitoring efforts by performing acoustic monitoring at spatial and temporal scales far beyond what is feasible for human observers (Blumstein et al. 2011). Compared with human point counts, acoustic monitoring with autonomous recording units can sample as many or more species in an equivalent sampling period (Darras et al. 2019) but can simultaneously sample hundreds of sites for months at a time, with each device sampling for multiple hours each day. As a result, the data sets generated by acoustic monitoring can quickly become so large that they are impossible to review manually. To address this challenge, scientists have developed automated recognition tools that enable the detection of species‐specific vocalizations in acoustic recordings with minimal manual labeling effort (Priyadarshani et al. 2018). In concert with automated acoustic recorders, automated recognition is currently being used to monitor biodiversity in novel ways. For instance, this technology has allowed researchers to evaluate the effectiveness of regional conservation plans for species that are otherwise difficult to observe, including bats (Weller 2008), forest elephants (Wrege et al. 2017), nocturnal birds (Shonfield et al. 2018), and cetaceans (Mellinger et al. 2007).

Various approaches have been proposed for automated identification of anurans in audio recordings, but few have been applied successfully to field data. Dutilleux and Curé (2018) built a custom signal processing model that accurately detected the European common spadefoot toad (*Pelobates fuscus*) in field data collected across multiple years and locations with underwater hydrophones, which record minimal background noise. Willacy et al. (2015) monitored the Richmond mountain frog (*Philoria richmondensis*) over thousands of hours of field data across multiple sites with a detector built with the proprietary Song Scope software (Wildlife Acoustics 2011) to identify the species with high accuracy. The authors reported that to prepare the Song Scope classifier, they spent 80 person‐hours tuning parameters and selecting vocalizations of the target species. Measey et al. (2017) used the software PAMGuard (PAMGuard 2020), which was developed for cetaceans, to monitor the Cape peninsula moss frog (*Arthroleptella lightfooti*) in a 4‐month study with six field recorders.

To date, two challenges have limited the use of automated recognition for detecting anurans. First, real‐world field recordings of anurans often contain background noise and heavily overlapping choruses of vocalizations from many individuals. For anurans in particular, large groups of vocalizing individuals can form choruses that sound very different from an isolated vocalization. Additionally, field data can span broad temporal and spatial scales, leading to variation in the vocalization of interest and the composition of background noise. For instance, ambient temperature influences the pitch and pulse repetition rate of many anuran calls (Narins 2001). A useful automated classifier must be able to provide accurate species identifications even in the presence of these complexities.

Second, automated recognition algorithms typically rely on a set of example recordings of the target species, known as training data, to train the recognition model. Many supervised machine learning classifiers for bats and birds, for example, are trained with hundreds of labeled audio clips of the target species (e.g., Priyadarshani et al. 2018). Such extensive training data, however, are not widely available for most anuran species. For example, only a fraction of amphibian species accounts on AmphibiaWeb (AmphibiaWeb 2020) has any audio recordings compared with virtually all bird species accounts on All About Birds (Cornell Lab of Ornithology 2020). Two alternatives to supervised machine learning classification may require less training data. Template‐based approaches (e.g., Lasseck 2013) were popular before deep learning became widely available, but they rely on stereotyped vocalizations and struggle to generalize (Huetz & Aubin 2012). Signal processing approaches (reviewed by Xie et al. 2018]) can recognize curated examples but have not been tested with field data, with the exception of one recognizer designed for one species (Dutilleux & Curé 2018).

**Figure 1:**
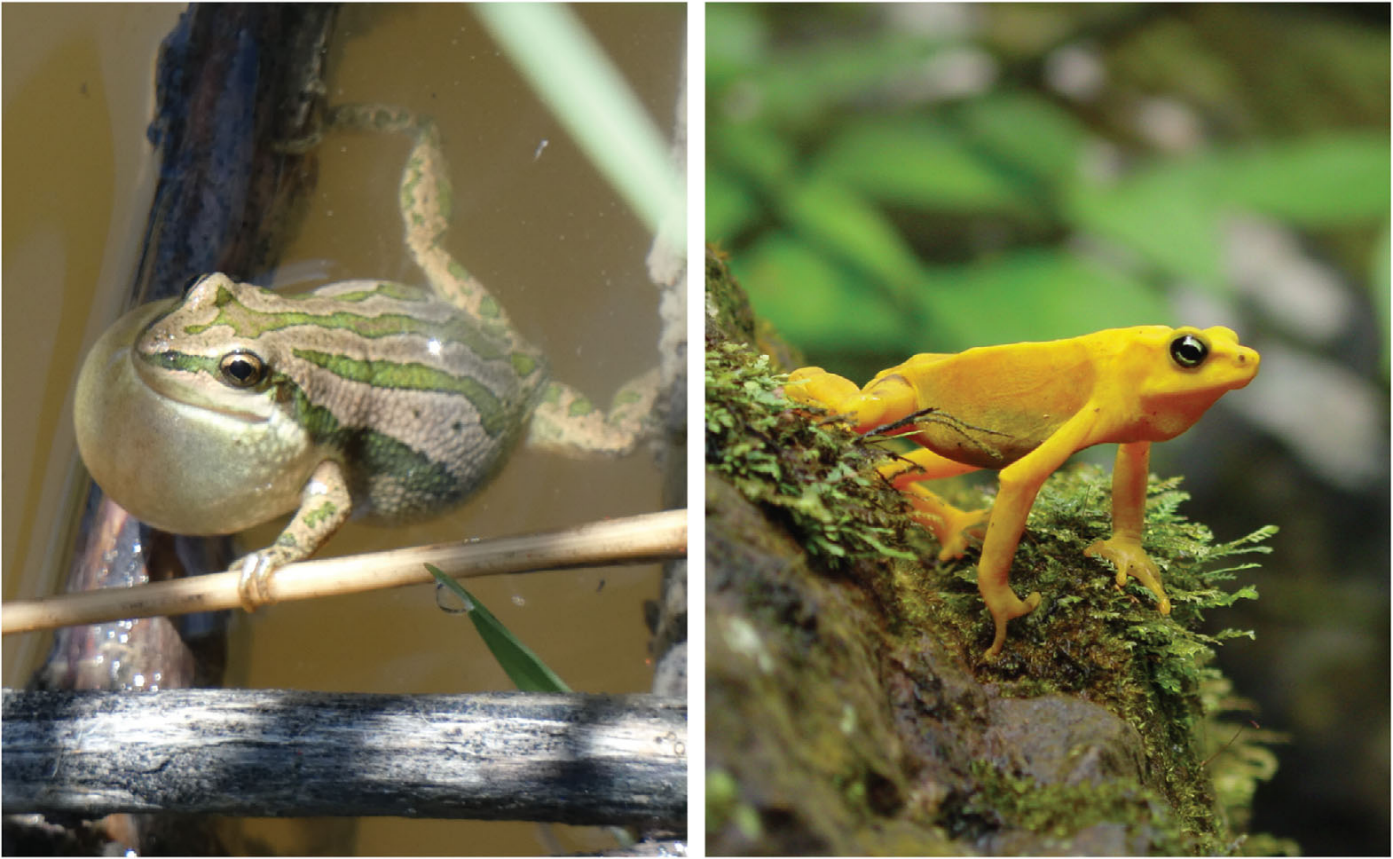
Boreal chorus frog (*Pseudacris maculata*) (left) and variable harlequin frog (*Atelopus varius*) (right)

We developed a new method for automated identification of anuran calls that can be applied to real‐world field recordings without requiring extensive training data. The repeat interval‐based bioacoustic identification tool (RIBBIT) is an open‐source detection and classification algorithm designed to detect vocalizations containing periodic structure. We applied this tool to detect vocalizations of the boreal chorus frog (*Pseudacris maculata*) (Figure 1) and critically endangered variable harlequin frog (*Atelopus varius*) (Figure 1). This tool provides an efficient method for monitoring populations of both species for conservation purposes, as well as a general framework for detecting and classifying vocalizations of other taxa with periodic structure.

## Methods

**Figure 2:**
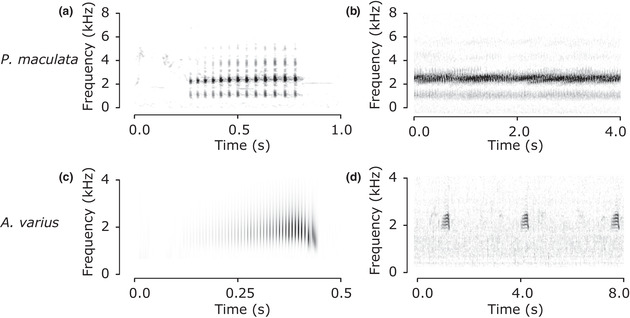
Spectrogram of a (a) boreal chorus frog call and (b) a chorus of many overlapping boreal chorus frog vocalizations, (c) scalogram (visual representation created from wavelets) of the rapid pulsing within a call of a variable harlequin frog, and (d) spectrogram of several consecutive variable harlequin frog calls. Graphs have different time and frequency scales to maximize resolution of features of interest

Anurans often produce calls with a periodic structure in which short elements within a call repeat at a consistent rate, called the pulse repetition rate (Ryan 2001). For example, a boreal chorus frog call has a pulse repetition rate of about 15 pulses per second (Figure 2a). We developed RIBBIT to detect such periodic acoustic signals occurring in specific frequency ranges.

**Figure 3:**
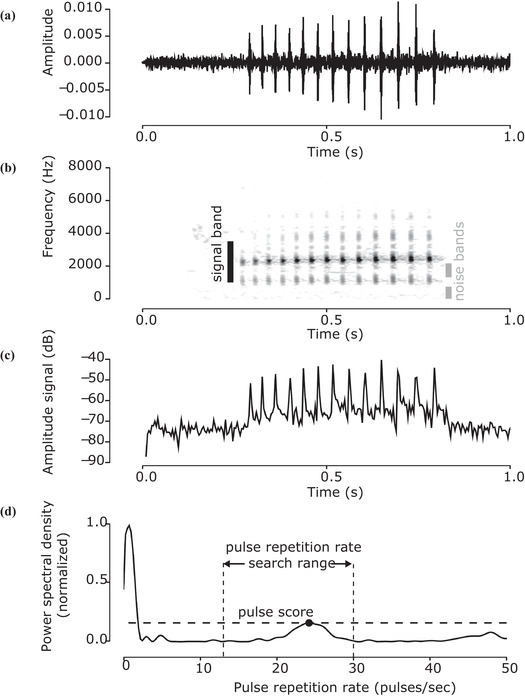
Calculation of RIBBIT score for boreal chorus frog in three steps: first, (a) the audio signal is converted into a spectrogram, which displays (b) frequency content versus time (the darker the pixel, the higher the value); second, values of the spectrogram inside the signal band are summed vertically and pixels in the noise bands are subtracted to create an (c) amplitude time signal; third, (d) the power spectral density of the amplitude signal is calculated. The RIBBIT score is the maximum value of the (unnormalized) power spectral density within the pulse repetition rate search range

We calculated a score for each audio clip using RIBBIT in three steps. First, we converted the audio signal (Figure 3a) into a spectrogram, a frequency versus time representation of audio created using the discrete Fourier transform (Figure 3b). Second, we created a summarized amplitude signal with one value for each column of the spectrogram (Figure 3c). Pixels in a preidentified signal band, containing the frequency range in which a species vocalizes, make positive contributions to this amplitude signal, whereas pixels in preidentified noise bands make negative contributions. Noise bands specify frequency ranges with undesired sounds, such as background noise, microphone pops and clicks, or the vocalizations of other species. Third, we measured the presence of periodic structure in the audio by calculating the power spectral density of the summarized amplitude signal (Figure 3d). The RIBBIT score is the maximum value of the power spectral density within a predefined range of pulse repetition rates. We ran RIBBIT on laptop computers and on a high‐performance computing cluster; typical computation speeds were 25–70 h of audio analyzed per core hour.

When possible, we assessed the performance of our models with precision, recall, and F1 scores. Precision indicates what fraction of the events receiving RIBBIT scores above a threshold actually contained the target vocalization. Recall indicates what fraction of all files that contained the target vocalization received scores above this threshold. The F1 score is the harmonic mean of the precision and recall scores and provides an indication of the overall accuracy of the model.

### Calculation of the RIBBIT score

Calculating a RIBBIT score involves creating a spectrogram from an audio file, reducing the spectrogram to a one‐dimensional amplitude signal, and calculating the maximum value of the power spectral density of that signal within a specified range of pulse repetition rates.

When creating a spectrogram, there is a trade‐off between frequency and time resolution, which is controlled by the spectrogram window size. For example, in case study 1 (described below) the audio has a 32 kHz sampling rate. We used 256 audio samples per window, with no overlapping samples between consecutive windows. Therefore, each vertical column of the resulting spectrogram represented 256 samples of audio with a sample rate of 32,000 samples per second, and therefore spanned a time period of 1/125 (32,000/256) seconds. This choice of spectrogram window length give sufficient temporal resolution to detect amplitude modulation up to 62.5 (125/2) pulses per second.

To better isolate the vocalization from other sounds in a recording, we defined a range of frequencies called the signal band containing the frequencies in which the focal species typically vocalizes. We also defined specific noise bands containing frequencies with undesired sounds. If the spectrogram is represented as an array of values across time and frequency $S_{f,t}$, then the amplitude signal is the per‐column sum of values in the signal band minus the values in the noise bands. With two noise bands, for example, this calculation is

(1) $$a_{\mathrm{net}}\left(t\right)\;=\;\alpha \sum_{f=f_{s0}\;}^{f_{s1}}S_{f,t}-\beta \sum_{f=f_{A0}\;}^{f_{A1}}S_{f,t}-\beta \sum_{f=f_{B0}\;}^{f_{B1}}S_{f,t},$$

where $f_{s0}$ and $f_{s1}$ are, respectively, the low and high frequencies of the signal band; $f_{A0}$ and $f_{A1}$ are, respectively, the low and high frequencies of noise band *A*; $f_{B0}$ and $f_{B1}$ are, respectively, the low and high frequencies of noise band *B*; and α and β normalize the contributions from the signal and noise band by frequency range so that the maximum possible contributions from the signal band and the combined noise bands are equal. The α and β were calculated, respectively, as

(2) $$\alpha =\frac{1}{f_{s1}-f_{s0}},$$

(3) $$\beta =\frac{1}{\left[\left(f_{A1}-f_{A0}\right)+\left(f_{B1}-f_{B0}\right)\right]}.$$

Negative values of the net amplitude signal, which occurred when the noise bands were stronger than the signal band, were set to zero to create the final amplitude signal.

Next, we measured the presence of periodic structure in the amplitude signal by calculating the power spectral density with Welch's method (Welch 1967). Given the minimum and maximum pulse repetition rates typical of the target species, $R_{l}$ and $R_{h}$, the RIBBIT score P is defined as the maximum value of the power spectral density within the range $\left[R_{l},R_{h}\right]$ (Figure 3d).

When calculating a RIBBIT score for long recordings, we divided audio files into segments of a fixed analysis window length with no overlap between windows. Each segment yielded a single *P*, and we called the sequence of RIBBIT scores for each segment start time the RIBBIT score sequence, P_ss_. Additional analysis, which we termed a continuity filter, can then be applied to the P_ss_ to return a positive detection only when multiple adjacent clips score above a chosen threshold. The continuity filter calculates a forward‐looking moving‐minimum ($P_{\mathrm{mm}}$) on P_ss_ over n segments:

(4) $$P_{\mathrm{mm}\;}\left(i\right)\;=\;\min\;\left[P_{\mathrm{ss}}\left(i\right),P_{\mathrm{ss}}\left(i+1\right),\ldots P_{\mathrm{ss}}\left(i+n-1\right)\right]\;,$$

where $P_{\mathrm{ss}}\left(i\right)$ selects *P* at each analysis window from $P_{\mathrm{ss}}\left(0\right)$ to $P_{\mathrm{ss}}\left(l-n\right)$ if $P_{\mathrm{ss}}$ has length l. We used this continuity filter in case study 1.

### Case study 1

In case study 1, our goal was to automatically detect calls of the boreal chorus frog. Chorus frogs often vocalize in large groups, producing a blended aggregate sound called a chorus (Figure 2b) that can be heard from hundreds of meters away (Garman 1892). These choruses often begin after dusk and can continue for hours. During the mating season, male boreal chorus frogs produce loud calls consisting of a series of slightly rising, wide‐band (1.5–6 kHz) pulses at a pulse repetition rate between 10 and 20 pulses per second (Figure 2a). Other anurans and birds at this site, including the great plains toad (*Anaxyrus cognatus*) and the Western meadowlark (*Sturnella neglecta*), sometimes produce vocalizations with a similar frequency range and pulse repetition rate.

We collected recordings from a mixed‐grass prairie ecosystem in north‐central Montana (USA) within the Northern Great Plains (48N, 108W) from private and public lands that are used mostly for cattle ranching operations. The long‐term goal of this research is to determine the effects of cattle and bison (*Bison bison*) grazing on the presence of five anuran species that breed in the region. We collected audio recordings from 23 sites during the boreal chorus frog breeding season in May and June of 2019 with AudioMoth recorders (Hill et al. 2019). We mounted recorders in plastic bags on rebar stakes, 50 cm above the ground, facing south. We set the devices to record at a 192 kHz sampling rate on medium gain and later down sampled audio to 32 kHz. The devices recorded three or six 10‐min segments (depending on the site) starting at dusk for seven consecutive days, which resulted in a data set of 819 ten‐minute files. To create a human‐annotated data set, we extracted the first 60 s of each 10‐minute file and reviewed each 60‐s file for boreal chorus frog vocalizations by simultaneously viewing the spectrogram and listening with closed‐back headphones.

Typical soundscapes recorded from this landscape are dominated by grassland birds, especially Western Meadowlark, Vesper Sparrow (*Pooecetes gramineus*), and Grasshopper Sparrow (*Ammodramus savannarum*) (Shamon, 2021). The two common sources of geophony are wind, which creates low‐frequency energy in recordings, and rain, which creates wide‐band pops when it strikes the recording device. We did not measure the environmental noise with a calibrated sound pressure meter. To approximate environmental noise levels, we first recorded 69 dBA pink noise with an AudioMoth on medium gain and measured the digital level to be −19 dBFS, calculated as $\mathrm{dBFS}=20\;\mathrm{lo}g_{10}\left(\sqrt{2}\;\cdot \mathrm{rms}\right)$, where rms is the root mean square value of the signal. This yielded a difference of −88 dB between the test signal in dbA and digital level in dbFS. We then calculated the digital level of our labeled files as −49 dBFS. Assuming very coarsely that the soundscape approximates pink noise, we estimated the average environmental noise to be around 40 dBA.

We selected the RIBBIT analysis parameters for boreal chorus frog detection (Table 1) through experimentation. An analysis window length of 2 s allowed sufficient temporal resolution while capturing entire calls. We found that using a signal band corresponding to the entire vocalizing range of boreal chorus frogs yielded many false positives, so we used a signal band of 1.0–3.3 kHz to target the strongest frequencies of the vocalization. Initial tests returned many files with broad‐band noises such as rain hitting the microphone and some great plains toad vocalizations. In addition to a low‐frequency noise band (100–1000 Hz), we used a second noise band within the signal band (1.8–2.5 kHz) to filter out the relatively narrow‐band vocalizations of great plains toads. Because vocalizations of a group of boreal chorus frogs normally last for 20 s or more, whereas similar vocalizations produced by grassland birds are shorter and separated by periods of silence, we used a continuity filter to detect only events with high scores for 10 consecutive 2‐s analysis windows.

**Table 1:** Parameters used for the repeat interval‐based bioacoustic identification tool (RIBBIT) analysis in case studies of boreal chorus frog and variable harlequin frog

Parameter	Unit	Symbol	Boreal chorus frog	Variable harlequin frog
Audio sampling rate	kHz		32	22.05
Spectrogram window length	Samples		256	32
Spectrogram window overlap	Samples		0	16
Signal band	Hz	$f_{s0}$, $f_{s1}$	1000, 3300	1500, 2500
Noise band 1	Hz	$f_{A0}$, $f_{A1}$	100, 1000	0,1000
Noise band 2	Hz	$f_{B0}$, $f_{B1}$	1800, 2500	3500,4000
Pulse rate (min, max)	Hz	$P_{l}$, $P_{h}$	13, 30	120, 150
Analysis window	s		2	0.5
Continuity filter	Windows	n	10	–

### Case study 2

In case study 2, we focused on the variable harlequin, a diurnal frog found in Panama and Costa Rica (Savage 1972; Pounds & Crump 1994). This species had been extirpated from large portions of its range due to human activity but remained common in less disturbed sites until the late 1980s. However, many populations crashed dramatically, beginning around 1987 in Costa Rica and 1999 in Panama (Crump & Pounds 1985; Pounds & Crump 1994; Lips et al. 2006), following the emergence and spread of the fungal disease chytridiomycosis (Berger et al. 1998; Longcore et al. 1999). These declines coincided with a larger pattern of disease‐induced extirpations of anurans in the region (Lips et al. 2006; Crawford et al. 2010) and around the world (Scheele et al. 2019). Many populations of the variable harlequin frog appeared to have disappeared completely by 1996 in Costa Rica (González‐Maya et al. 2013). In some parts of Panama, populations declined precipitously following disease outbreaks (Crawford et al. 2010), and the species has been observed infrequently since (González‐Maya et al. 2013; Perez et al. 2014; Voyles et al. 2018). This species is currently considered critically endangered (Pounds et al. 2010).

Male variable harlequin frogs call from streamside territories during the daytime, producing a simple croak‐like call repeated at semiregular intervals of about 3.5 s (Figure 2d). The call has the most energy between 1500 and 2200 Hz and consists of rapid amplitude modulation at a pulse repetition rate of around 130 pulses per second (Figure 2c) (Cocroft et al. 1990). This extremely rapid pulsing is perceived by human listeners as a continuous tone. We show a scalogram in Figure 2c because it provides higher time resolution than a spectrogram, but only spectrograms were used during analysis.

For this case study, we deployed AudioMoths to record at three sites in Panama. We selected the three sites based on previous observations of individual variable harlequin frogs following disease‐induced declines (Perez et al. 2014; Voyles et al. 2018): two sites near the town of El Copé, Coclé Province, including inside the G. D. Omar Torrijos National Park, and one site near the town of Santa Fe, Veraaguas Province, near Santa Fe National Park. These areas are tropical moist forests with a dry season lasting from mid‐December to mid‐April and a wet season lasting from mid‐April to mid‐December. At each site, we placed eight to 10 AudioMoth recorders in plastic bags on a streamside transect with 10‐ to 20‐m spacing between devices. We placed AudioMoths on trees within 1.5 m of the stream and approximately 0.5–1.5 m above the water or ground. We programed the devices to record on medium gain for 55 min/h, 24 h a day, for three consecutive days during one wet season (August 2019) and one dry season (December 2019). This approach generated 2345 h of audio. We resampled all recordings to 22 kHz and split all files into 60‐s clips.

Typical daytime soundscapes recorded from this deployment are dominated by stream noise, which masks all but the nearest animal vocalizations. The sites featured high biodiversity with as many as 60 amphibian species (Crawford et al. 2010). We manually reviewed a random sample of 300 ten‐second files from the field data and found no vocalizations of variable harlequin frogs. Using the same procedure as in case study 1, we estimated the average noise level in the soundscapes as approximately 65 dBA based on a random sample of 1000 sixty‐second files.

Before analyzing the field data, we had just one recording of a variable harlequin frog vocalization collected during prior research. We ran RIBBIT to search for amplitude modulation between 120 and 150 pulses per second (parameters in Table 1). We chose a short analysis window of 0.5 s because the call duration was approximately 0.3 s. To achieve the time resolution necessary to resolve 130 pulses per second, we used a spectrogram window size of only 32 samples.

## Results

### Case study 1

**Figure 4:**
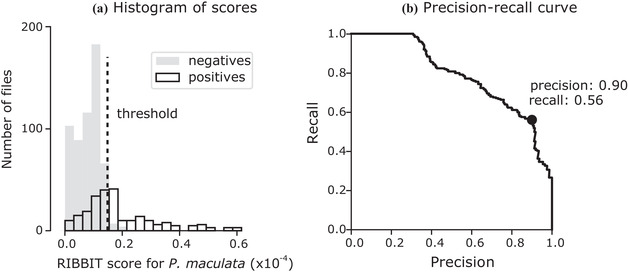
(a) The RIBBIT scores for labeled files that contained boreal chorus frog vocalizations and those that did not. Three positive files scored above 0.6 and are not shown. (b) Precision–recall curve showing the trade‐off between high precision (few false positives) and high recall (few false negatives) (dot, precision and recall at the selected threshold of 1.48 × 10^−5^)

We successfully detected boreal chorus frogs in the American Prairie field data with RIBBIT. In a manual review of the selected data, we found chorus frog vocalizations in 249 of the 819 files. For the classifier, the choice of a threshold score controlled the trade‐off between high precision (few false positives) and high recall (few false negatives) (Figure 4b). A histogram of RIBBIT scores for the labeled files that contained boreal chorus frog vocalizations (positives) and those that did not (negatives) revealed a natural threshold for determining the presence of boreal chorus frogs of 1.48 × 10^−5^ (Figure 4a). At this threshold, the model achieved a precision of 0.90, a recall of 0.56, and an $F_{1}$ score of 0.69 on the human‐annotated files. The model generally scored background noise, rain hitting the microphone, and vocalizations of confusion species far below this threshold.

### Case study 2

**Figure 5:**
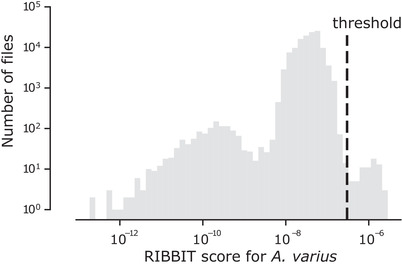
The RIBBIT scores for each 60‐s file in the Panama field data

We were surprised to successfully detect variable harlequin frog vocalizations in the field recordings from Panama. With a logarithmic scale on both axes, the distribution of RIBBIT scores for the 60‐s files appears to have three distinct parts, with a natural break between the highest and middle parts at a threshold of 3  ×  10^−7^ (Figure 5). Of the over 120,000 audio clips, 72 scored above this threshold. Seventy of these 72 files contained variable harlequin frog vocalizations, for a precision of 0.97 at this threshold. These 70 audio files are publicly available (see DATA AVAILABILITY). All of the detected variable harlequin frog vocalizations were captured by the same AudioMoth recorder during one 3‐day deployment and may have been produced by a single individual. We manually inspected a small set of files from the centers of the middle and lower parts of the score distribution and found that the lowest part contained mostly silent files, whereas the middle part contained mostly files with stream noise. Although the field sites contain considerable anuran diversity, the model consistently gave low scores to files that contained biotic noise such as other frogs, insects, and birds and abiotic noise such as rain and water.

## Discussion

In case study 1, we demonstrated that our RIBBIT method accurately detected the vocalizations of a common North American frog species, even in the presence of several common challenges associated with real‐world field data. Using RIBBIT, we were able to detect boreal chorus frogs with high precision (0.90) and moderate recall (0.56), despite the prevalence of heavily overlapping choruses, background noise, and calls of other species with similar structure. Using the continuity filter effectively reduced false positives from similar‐sounding species such as the Western meadowlark, but also caused false negatives for isolated boreal chorus frog vocalizations separated by seconds of silence.

In case study 2, we demonstrated that RIBBIT detected vocalizations of a rare and endangered species when only a single human‐annotated training file was available. This detection was particularly surprising because annual human transect surveys since 2012 have recorded only one observation of a single individual variable harlequin frog at this site (J.V., personal observation). Because we did not have labeled data for this case study, we could not measure the recall of the classifier and therefore could not estimate how many variable harlequin frogs may have been missed. Additionally, because the detected vocalizations occurred at a single date and site, it is possible that our reported performance may reflect the call structure of one or few individual variable harlequin frogs. A larger dataset with vocalizations of many individuals would be necessary to determine the robustness of these quantitative results to variation in call structure across individuals.

RIBBIT will be most useful in future applications when the target vocalization and environment meet three basic conditions. First, the target vocalization should contain regularly repeated pulses or calls, such as those produced by many anurans (Ryan 2001) as well as some birds and insects. For some taxa including anurans, pulse repetition rate may depend in part on temperature (Ryan 2001). For these species, the RIBBIT parameters should be chosen to include the full range of pulse repetition rates that may occur given the temperature range at a site. If ambient temperature data are available, these could be used to tighten this range, potentially improving model performance. Second, the combination of signal band and pulse repetition rate of the target vocalization should be unique among sounds present in the environment. If two species produce vocalizations with the same signal band and pulse repetition rates, the model has no way of differentiating them at the level of a single file. For example, in case study 1 the RIBBIT model could not reliably differentiate boreal chorus frog calls from some elements of the Western Meadowlark song, which led us to use a continuity filter to reject Western Meadowlark songs. Third, sufficient information must be available to select a signal band, noise bands, and pulse repetition rate for the target vocalization. These may be drawn from as little as one annotated audio recording of the vocalization or may come from expert knowledge.

Future work could expand on our methods in three specific ways to create even more robust and generalizable automated recognition methods. First, the pulse rate recognition strategy used by RIBBIT may be generalized by applying nonlinear transformations to the time domain of the signal so that systematically nonperiodic temporal patterns can be recognized. For example, in pilot experiments, we were able to detect the accelerating drumming display of the Ruffed Grouse (*Bonasa umbellus*) in audio recordings by applying a polynomial transformation to the amplitude signal. Second, further work could leverage alternative representations of audio data. When creating a spectrogram, there is a well‐known trade‐off between frequency resolution and time resolution. Wavelet analysis can create an alternative representation of audio that overcomes this frequency and time resolution trade‐off (Lang & Forinash 1998). We used spectrograms in this work because spectrograms provide sufficient resolution and wavelet analysis is more computationally expensive. However, future studies could use wavelet analysis if more frequency and time resolution is required. Third, additional acoustic features could be used to augment the RIBBIT score. Including other features, such as spectral peak tracts, mel‐frequency cepstral coefficients, syllable duration, and spectral energy measurements, alongside pulse repetition rate could lead to more robust automated recognition tools (Xie et al. 2018). In particular, because the pulse repetition rate focuses heavily on temporal information, features that characterize the spectral characteristics of a vocalization could complement this method.

To aid future investigators in applying RIBBIT to other communities, we have provided open‐source implementations of RIBBIT in Python and R under the MIT license (see DATA AVAILABILITY).

In summary, we believe that RIBBIT has the potential to be a broadly useful tool for automated recognition in anuran conservation research. In two case studies, the method was specifically robust to three challenging characteristics of real‐world anuran field recordings: heavily overlapping choruses, background noise, and other species with similar vocalizations. In addition, unlike many supervised machine learning classifiers, RIBBIT does not require extensive training data. In fact, with as little as one example recording, an investigator could try using RIBBIT to detect any vocalization with a periodic amplitude fluctuation.

Given the global scale of amphibian declines, many of which have occurred in remote areas of the tropics, the ability to identify sites where rare and imperiled species persist from recordings has the potential to rapidly improve understanding of threats, such as emerging diseases (Scheele et al. 2019) and climate change (Cohen et al. 2019), and to aid in conservation efforts to protect these species. Acoustic monitoring can allow biologists to collect massive amounts of audio recordings from ecosystems around the globe, but investigators will need automated recognition tools to help transform these recordings into species‐specific observations. In combination with acoustic monitoring, we believe that tools such as RIBBIT have the potential to support worldwide efforts to mitigate ongoing losses of amphibian biodiversity.

## Data Availability

The 70 audio files of *A. varius* vocalizations are publicly available from Dryad (Kitzes et al. 2021). The Python implementation of RIBBIT is in the open‐source bioacoustics software package OpenSoundscape (Kitzes et al. 2020). The R implementation of RIBBIT is in an R markdown notebook available on github (Lapp 2020a). Python and R notebooks containing example analyses for each case study are also available on github (Lapp 2020b).
